# Antioxidant vitamin intake and mortality in three Central and Eastern European urban populations: the HAPIEE study

**DOI:** 10.1007/s00394-015-0871-8

**Published:** 2015-03-12

**Authors:** Urszula Stepaniak, Agnieszka Micek, Giuseppe Grosso, Denes Stefler, Roman Topor-Madry, Ruzena Kubinova, Sofia Malyutina, Anne Peasey, Hynek Pikhart, Yuri Nikitin, Martin Bobak, Andrzej Pająk

**Affiliations:** 1 Department of Epidemiology and Population Studies, Institute of Public Health, Faculty of Health Sciences, Jagiellonian University Medical College, Grzegorzecka Street 20, Krakow, 31-531 Poland; 2 Department of Clinical and Molecular Biomedicine, Section of Pharmacology and Biochemistry, University of Catania, Catania, Italy; 3 Department of Epidemiology and Public Health, University College London, London, UK; 4 National Institute of Public Health, Prague, Czech Republic; 5 Institute of Internal and Preventive Medicine Siberian Branch Under the Russian Academy of Medical Sciences, Novosibirsk, Russia; 6 Novosibirsk State Medical University, Novosibirsk, Russia

**Keywords:** Antioxidant vitamin, Mortality, Cardiovascular, Central and Eastern Europe

## Abstract

**Purpose:**

The aim of the study was to assess the relationships between individual-level dietary intakes of antioxidant vitamins C, E and beta-carotene with all-cause and cause-specific mortality in three Central and Eastern European (CEE) populations.

**Methods:**

Data from the Health, Alcohol and Psychosocial factors in Eastern Europe cohort study were used. At the baseline survey, between 2002 and 2005, 28,945 men and women aged 45–69 years were examined in Novosibirsk (Russia), Krakow (Poland) and seven Czech towns. Deaths in the cohorts were identified through mortality registers. Cox regression was used to estimate the association between vitamin consumption and all-cause, cardiovascular (CVD) disease and cancer mortality.

**Results:**

In multivariable-adjusted analyses, there were no clear inverse associations between antioxidant vitamin intakes and mortality, although in some groups, several hazard ratios (HRs) were significant. For example, in men, compared with the lowest quintile of vitamin C intake, all-cause mortality in the third and fourth quintiles was lower by 28 % (HR 0.72; 95 % CI 0.61–0.85) and by 20 % (HR 0.80; 95 % CI 0.68–0.95), respectively. CVD mortality was lower by 35 % (HR 0.65; 95 % CI 0.50–0.84) and by 23 % (HR 0.77; 95 % CI 0.59–0.99) in third and fourth quintile of vitamin C intake, respectively. In women, the third and fourth quintiles of dietary intake of vitamin E were associated with reduced risk of all-cause death by 33 % (HR 0.67; 95 % CI 0.53–0.84) and by 23 % (HR 0.77; 95 % CI 0.61–0.97), respectively. Consumption of vitamin C, vitamin E and beta-carotene was not related to CVD mortality in women and to cancer mortality in either gender.

**Conclusion:**

This large prospective cohort study in CEE populations with low prevalence of vitamin supplementation did not find a strong, dose–response evidence for protective effects of antioxidant vitamin intake.

**Electronic supplementary material:**

The online version of this article (doi:10.1007/s00394-015-0871-8) contains supplementary material, which is available to authorized users.

## Introduction

Mortality in Central and Eastern European (CEE) countries decreased in the last two decades, but it is still higher than in Western Europe [1]. Since the main causes of deaths are CVD and cancer, modifiable risk factors, such as dietary habits, may play an important role in the etiology of chronic diseases and their association with survival. Socioeconomic transition in CEE countries in the 1990s caused significant changes in food consumption, which may have an impact on health and life expectancy [2]. Antioxidants have been hypothesized to extend the lifespan [3]. Vitamin C, vitamin E and provitamin A carotenoids (primarily beta-carotene), which are essential antioxidants in diet, may prevent oxidative damages by counteracting the effects of free radicals [4–6]. Antioxidant vitamins are present in a variety of foods. Fruits, particularly citrus fruits, and vegetables are rich in vitamin C. Most dietary beta-carotene comes from orange and yellow vegetables, dark green leafy vegetables, fruits and some vegetable oils. Vitamin E is found in vegetable oils, nuts, wheat germ, seeds, green leafy vegetables, and also eggs, milk and meat and their products [7–9]. Despite dietary patterns based on foods with high antioxidant contents having been demonstrated to exert beneficial effects on human health [10, 11], most interventional studies found no association between vitamin supplements and mortality [12, 13]. In observational studies, high dietary intake of antioxidant vitamins has been associated with decreased risk of all-cause [7, 14, 15] and CVD death [7, 8, 15–18], but this relationship was not confirmed in all studies [19–22]. Evidence on the relationship between the intake of antioxidant vitamins and the risk of cancer is ambiguous [7, 19, 23, 24]. The inconsistent results may be due to different methods of vitamin intake assessment, large within- and between-individual variation in nutrient intake, differences in the age of participants or the variation in confounding factors related to social and lifestyle characteristics of study subjects considered [25].

It is possible that the association between vitamin intake and potential confounders differs by societies, so analysis in a variety of social contexts could help to understand the relation between natural vitamins intake and mortality. In CEE populations, the structure of potential confounders is different than in most previous observational studies in economically advanced populations of Western Europe and other parts of the world. For example, in CEE, the proportion of economically disadvantaged people is higher than in Western Europe, and the prevalence of vitamin supplementation is lower [26, 27]. Additionally, exposure to other lifestyle risk factors, such as smoking or alcohol drinking, is different compared to Western Europe [28, 29].

Only a few cross-sectional and ecological studies have explored dietary antioxidant consumption in CEE countries, [2, 26, 27, 30–32] and there is no evidence from prospective studies of an association between the individual dietary consumption of antioxidant vitamins and mortality in this region. The aim of the present study was to assess the relationship between dietary intake of antioxidant vitamins C, E and beta-carotene with all-cause, CVD and cancer mortality in three CEE urban population samples.

## Materials and methods

### Study populations

We used data from baseline examination and mortality follow-up of the Health, Alcohol and Psychosocial factors in Eastern Europe (HAPIEE) study, which was conducted in Novosibirsk (Russia), Krakow (Poland) and seven Czech towns (Havirov/Karvina, Jihlava, Usti nad Labem, Liberec, Hradec Kralove and Kromeriz). Details of the study design have been published elsewhere [33]. At baseline, random samples of men and women aged 45–69 years, stratified by gender and 5-year age groups, were selected from urban population registers (electoral lists in Russia). In Krakow and Czech towns, subjects were first visited at home to complete questionnaire and then invited to a clinic for examination; in Novosibirsk, both the questionnaires and examinations were completed in a clinic. In total, 28,945 respondents took part in the baseline survey, and the overall response rate was 59 % [33]. The study was approved by the University College London Hospital Ethics Committee and by the local ethics committee in each participating center. All participants gave written informed consent.

### Measurements of vitamin intake

Individual dietary intake of vitamin C, vitamin E, beta-carotene and energy was calculated based on a standard food frequency questionnaire (FFQ) [2]. FFQs consisted of 136, 147 and 148 food and drink items, in Czech towns, Novosibirsk and Krakow, respectively. Reliability of local version of the questionnaire was checked by back translation into English [33]. Country-specific portion size for each food was specified, and participants were asked how often, on average, they had consumed that amount of a particular food, with nine responses ranging from “six or more times per day” to “never or less than once per month”. In the next step, frequency of food consumption was converted into daily food consumption, and daily intake of energy and nutrients was then calculated by multiplying the frequency of food consumed per day by the nutrient content of the specified standard portion [2]. The validity of the FFQ regarding micronutrient intake data was assessed by estimating correlations with plasma biomarker concentrations measured in a central laboratory (CTSU, Oxford, UK) in a random subsample of participants. The correlations for vitamin C and beta-carotene were similar to other published large-scale studies [34]. Regarding vitamin E, the energy-adjusted Pearson’s partial correlation coefficient between vitamin E intake and alpha-tocopherol plasma level was 0.15 in the pooled sample, which was slightly lower than the average of 21 previously published similar studies [35].

For the present analysis, we excluded 709 individuals with invalid diet data (defined as having missing data on more than 50 % of food items or reported extreme values of energy intake: <500 kcal/day/>4500 kcal/day in women; <800 kcal/day/>5000 kcal/day in men). General information on supplementation with vitamins and minerals was collected based on the questions: “Do you take any vitamins or mineral supplements?” and “If yes, do these supplements contain vitamin C?” Those who declared to use supplements containing vitamin C regularly, at least three times per week, were classified as users of supplements of vitamin C.

### Mortality

Data on deaths were obtained by linkage with mortality registers. In the Krakow cohort, 716 (6.7 %) individuals at baseline did not agree for prospective observation and were not followed up. For the present analysis, the follow-up for vital status was complete until December 31 2010 in Krakow and Novosibirsk and until December 31 2011 for Czech towns. Median follow-up time was 8.1 years in Czech towns, 6.5 years in Novosibirsk and 7.1 years in Krakow. Cause-specific mortality was based on the ICD 10th revision: I00−I99 for CVD deaths and C00−C97 for cancer death. For 527 persons, no information on follow-up time was available, and they were excluded in this analysis. The final sample included in the analysis consisted of 26,993 individuals, of whom 2371 died during the follow-up period, including 997 CVD deaths and 830 cancer deaths.

### Potential confounders

At baseline examination, a standard questionnaire was used to collect data on age, gender, education (categorized into three groups: university, secondary and lower), current smoking (yes/no) and alcohol consumption during the last 12 months (g/day), health status (self-reported history of myocardial infarction, stroke, cancer and diabetes). Hypertension was defined as systolic blood pressure ≥140 or diastolic blood pressure ≥90 mmHg or taking antihypertensive drugs. Based on standardized measurements of body weight and height, body mass index (BMI, kg/m^2^) was calculated and categorized into three groups: normal (BMI < 25.0), overweight (BMI 25.0–29.9) and obese (BMI ≥ 30.0). Hypercholesterolemia was defined as having serum total cholesterol level ≥5 mmol/l.

### Statistical analysis

Association between intake of antioxidant vitamins and all-cause and cause-specific mortality was evaluated by Cox proportional hazards regression models. Person-years of observation were calculated from the date of baseline examination to the date of death or the end of follow-up, whichever came first. Individuals who were lost to follow-up were censored at the last date of contact. The proportional hazards assumptions in Cox models were checked using Schoenfeld’s test examining interactions with log-transformed time to the event and indicating no significant violation of the assumption [36]. Country- and gender-specific quintiles of energy-adjusted intake of vitamins C, E and beta-carotene were made. Dietary intakes of vitamins were adjusted for total energy intake using the residual method [37]. Bottom quintile of vitamin intake was used as the reference category. First, analyses were made separately by country, and the associations between vitamin intakes and mortality were similar across the countries. Then, the interaction between quintiles of each antioxidant vitamin intake and countries was assessed by likelihood ratio test, comparing models with and without the interaction terms. Significant interactions were detected for men in the models testing influence of beta-carotene intake on the risk of both all-cause and CVD mortality and for women in the model testing influence of vitamin C intake on the risk of cancer mortality. In all other cases, no significant interaction was observed, and the data from the three centers were pooled. In the pooled data, two gender-specific Cox models were used: first adjusted for age and country; second additionally adjusted for other potential confounders (education, smoking status, alcohol intake, BMI, hypertension, diabetes, hypercholesterolemia, history of CVD or cancer and total energy intake).

For cause-specific mortality, individuals who died from causes other than those investigated were censored at the date of death. Additionally, we fitted models in which death from causes other than those investigated was treated as a competing risk. These models showed similar associations as those observed in non-competing risk models for deaths from cardiovascular diseases and cancer, so we did not present the results in the tables. Differences in the distributions of the studied characteristics between quintiles of vitamin intake were tested by *χ* ^2^ test for categorical variables. For continuous variables with normal and non-normal distribution, ANOVA and Kruskal–Wallis tests were used, respectively. To deal with the potential effect of reverse causality, additional models were run after excluding individuals who died within the first 2 years of follow-up and after excluding participants with history of myocardial infarction or stroke or cancer at baseline. Also, models after exclusion of users of vitamin C supplements were constructed. Statistica version 10.0 and R version 3.0.2. software were used; *p* < 0.05 was set as the level of statistical significance.

## Results

Baseline characteristics of the studied sample by gender and country are presented in Table 1. During follow-up, the number of total deaths was 1590 (12.6 %) in men and 781 (5.4 %) in women. Number of CVD and cancer deaths was 684 (5.4 %) and 527 (4.2 %) in men and 313 (2.2 %) and 303 (2.1 %) in women, respectively. Total energy intake and beta-carotene intake were the highest in Novosibirsk and the lowest in Czech towns, while intake of vitamin C was the lowest in Novosibirsk and the highest in Czech towns. The lowest vitamin E intake was observed in Krakow. The proportion of participants with university education was similar in Novosibirsk and Krakow and lower in Czech towns. In Novosibirsk, the proportion of smoking men was the highest, and the proportion of smoking women was the lowest. Russian sample differed from the others by having the lowest frequency of diabetes, history of cancer, users of vitamin C supplement, but the highest frequency of history of CVD.

**Table 1:** Baseline descriptive statistics and number of deaths by gender and population

Baseline characteristics	Czech towns		Novosibirsk		Krakow	
	Men *n* = 3793	Women *n* = 4366	Men *n* = 4074	Women *n* = 4946	Men *n* = 4775	Women *n* = 5039
Age, mean (SD)	58.6 (7.2)	57.9 (7.1)	58.4 (7.0)	58.1 (7.1)	57.9 (7.0)	57.4 (7.0)
Education (%)						
Primary/vocational	49.8	48.8	33.3	40.3	36.6	28.6
Secondary	31.8	41.1	34.7	33.2	33.4	44.4
University	18.4	10.1	32.0	26.5	30.0	27.1
Current smokers (%)	26.7	20.7	48.8	9.2	33.8	26.0
BMI (kg/m^2^), mean (SD)	28.3 (4.0)	28.1 (5.0)	26.6 (4.4)	30.2 (5.7)	28.0 (4.0)	28.4 (5.1)
Alcohol (g/day), median (q1–q3)	8.5 (1.4–27.3)	0.8 (0.1–3.9)	6.6 (1.2–21.0)	0.7 (0.1–1.1)	2.2 (0.1–8.6)	0.7 (0.1–1.1)
Hypertension (%)	72.9	58.3	63.2	67.0	66.3	55.9
Total cholesterol (mmol/l), mean (SD)	5.6 (1.0)	5.8 (1.0)	6.0 (1.2)	6.5 (1.3)	5.7 (1.1)	5.9 (1.1)
Diabetes (%)	13.3	10.2	4.1	6.1	13.6	10.1
History of MI or stroke (%)	10.6	4.6	14.7	8.6	13.0	6.1
History of cancer (%)	4.2	8.2	1.3	4.0	3.3	6.2
Total energy intake (kcal/day), mean (SD)	2081.3 (670.8)	1940.4 (662.2)	2742.8 (772.7)	2335.0 (688.8)	2259.9 (680.9)	2046.8 (611.5)
Use of supplements with vitamin C (%)	14.1	23.0	5.9	14.4	9.9	16.8
Vitamin C (mg/day), median (q1–q3)	134.4 (90.7–196.6)	189.0 (123.4–273.4)	71.2 (52.5–102.4)	89.7 (62.9–136.9)	123.9 (89.9–174.1)	144.0 (101.1–204.1)
Vitamin E (mg/day), median (q1–q3)	8.3 (6.9–10.0)	9.9 (8.2–11.8)	9.8 (8.2–11.7)	11.4 (9.6–13.6)	7.7 (6.5–9.0)	8.1 (6.9–9.4)
Beta-carotene (ug/day), median (q1–q3)	4528.5 (3295.7–6358.9)	5560.3 (3965.3–8969.4)	8662.3 (5770.5–11,545.9)	10,824.7 (7459.1–13,684.2)	6918 (4515.9–9330.3)	8037.5 (5308.9–10,908.8)
Follow-up time (years), mean (SD)	7.9 (1.6)	8.1 (1.2)	6.0 (1.6)	6.5 (1.1)	6.9 (1.3)	7.1 (1.0)
Number of deaths (%)	447 (11.8)	243 (5.6)	615 (15.1)	255 (5.2)	528 (11.1)	283 (5.6)
Number of CVD deaths (%)	166 (4.4)	83 (1.9)	333 (8.2)	135 (2.7)	185 (3.9)	95 (1.9)
Number of cancer deaths (%)	186 (4.9)	115 (2.6)	145 (3.6)	74 (1.5)	196 (4.1)	114 (2.3)

Distribution of gender-specific baseline characteristics across quintiles of vitamin C, vitamin E and beta-carotene intakes is presented in Table 2. In men, high dietary intake of antioxidant vitamins was related to higher age, higher education, lower prevalence of smoking and alcohol consumption, lower caloric intake, use of vitamin C supplements and more frequent health problems (hypertension, diabetes and history of CVD). History of cancer in men was related to vitamin C intake but inversely related to vitamin E and beta-carotene intake. Women with high vitamin C intake were younger, better educated, more likely to consume alcohol, consume more calories and vitamin C supplements, healthier and had lower BMI, while women with high vitamin E and beta-carotene intake were older, less likely to smoke and drink alcohol and more likely to have health problems.

**Table 2:** Distribution of baseline characteristics and number of deaths according to quintiles of vitamin intake by sex

Characteristics	Quintiles of vitamin C intake (mg/day)						Quintiles of vitamin E intake (mg/day)					
	1 (low)	2	3	4	5 (high)	*p*	1 (low)	2	3	4	5 (high)	*p*
*Men*												
Median of vitamin intake	51.1	78.4	107.7	148.8	231.3	<0.001	5.9	7.3	8.5	9.8	12.2	<0.001
No. of subjects	2528	2528	2530	2527	2529	–	2531	2527	2533	2524	2527	–
No. of all-cause deaths	422	339	247	267	315	<0.001	288	290	299	324	389	<0.001
No. of CVD deaths	206	157	93	99	129	<0.001	108	109	118	145	204	<0.001
No. of cancer deaths	112	103	88	101	123	ns	112	109	106	97	103	ns
Age, mean (SD)	58.4 (7.1)	58.1 (6.9)	58.0 (7.0)	58.2 (7.1)	58.6 (7.1)	<0.05	57.8 (7.0)	58.0 (7.0)	58.2 (7.1)	58.3 (7.1)	59.0 (7.0)	<0.001
Education (%)												
Lower	41.6	39.9	38.6	38.9	28.3	<0.001	41.1	40.6	37.3	38.7	39.7	<0.05
Secondary	36.3	32.7.	33.1	32.0	32.6		32.8	34.1	34.7	33.3	31.8	
University	22.1	274	28.3	29.1	29.1		26.2	25.3	28.0	28.0	28.5	
Current smokers (%)	51.5	41.0	34.3	29.1	26.8	<0.001	38.7	34.8	35.1	37.4	36.6	<0.05
BMI (kg/m^2^), mean (SD)	26.5 (4.4)	27.4 (4.3)	27.8 (4.0)	28.0 (4.0)	28.4 (4.1)	<0.001	27.7 (3.9)	27.8 (4.1)	27.7 (4.3)	27.4 (4.3)	27.4 (4.5)	<0.01
Alcohol (g/day), median (q1–q3)	6.6 (1.0–21.9)	5.0 (0.7–17.2)	4.5 (0.7–14.9)	4.4 (0.7–14.9)	3.8 (0.7–14.9)	<0.001	5.0 (0.6–20.9)	5.0 (0.9–17.1)	5.0 (0.7–15.7)	5.0 (0.8–15,8)	4.8 (0.7–15.4)	ns
Total energy intake (kcal/day), mean (SD)	2436.7 (835.3)	2386.3 (774.2)	2348.3 (751.4)	2305.8 (709.4)	2333.0 (713.8)	<0.001	2392.2 (930.3)	2378.8 (788.0)	2345.7 (721.9)	2372.8 (687.7)	2320.0 (632.8)	<0.01
Use of supplements with vitamin C (%)	5.8	7.9	9.3	10.4	15.9	<0.001	13.0	12.6	13.5	12.9	11.9	ns
Hypertension (%)	62.7	65.9	66.7	68.6	71.6	<0.001	66.3	66.0	66.5	66.4	69.7	<0.05
Total cholesterol (mmol/l), mean (SD)	5.9 (1.2)	5.9 (1.2)	5.8 (1.1)	5.7 (1.1)	5.7 (1.1)	<0.001	5.8 (1.1)	5.8 (1.1)	5.8 (1.1)	5.8 (1.1)	5.8 (1.2)	ns
Diabetes (%)	6.4	8.6	10.3	12.5	14.4	<0.001	9.3	10.2	9.9	10.6	12.1	<0.05
History of CVD (%)	14.5	13.4	12.0	11.1	13.3	<0.01	10.9	11.4	12.9	12.9	16.3	<0.001
History of cancer (%)	2.1	2.6	2.7	3.8	3.3	<0.01	3.4	3.5	2.5	2.7	2.5	ns
*Women*												
Median of vitamin intake	59.3	94.7	133.8	189.6	308.2	<0.001	6.6	8.3	9.6	11.2	14.1	<0.001
No. of subjects	2871	2870	2869	2870	2871	–	2868	2873	2869	2867	2874	–
No. of all-cause deaths	187	145	162	144	143	<0.05	196	154	126	140	165	<0.001
No. of CVD deaths	94	56	60	52	51	<0.001	68	57	60	56	72	ns
No. of cancer deaths	59	55	62	59	68	ns	74	60	50	57	62	ns
Age, mean (SD)	58.7 (7.3)	57.8 (7.1)	57.4 (7.0)	57.4 (7.0)	57.5 (6.9)	<0.001	57.7 (7.2)	57.5 (7.0)	57.7 (7.0)	57.7 (7.0)	58.3 (7.2)	<0.001
Education (%)												
Lower	44.0	39.6	37.8	35.4	37.0	<0.001	35.7	37.4	37.7	39.1	43.9	<0.001
Secondary	37.3	38.2	40.0	40.9	41.3		41.6	40.5	40.9	39.7	35.0	
University	18.7	22.3	22.2	23.7	21.6		22.7	22.1	21.4	21.2	21.1	
Current smokers (%)	16.3	18.9	20.1	19.0	18.7	<0.01	24.9	21.0	18.6	16.3	12.2	<0.001
BMI (kg/m^2^), mean (SD)	29.5 (5.8)	29.2 (5.5)	29.0 (5.2)	28.6 (5.2)	28.6 (5.2)	<0.001	28.1 (5.1)	28.6 (5.2)	28.9 (5.4)	29.2 (5.4)	30.0 (5.7)	<0.001
Alcohol (g/day), median (q1–q3)	0.3 (0.0–1.1)	0.3 (0.0–1.3)	0.3 (0.0–1.7)	0.4 (0.0–2.3)	0.4 (0.0–2.3)	<0.001	0.2 (0.0–1.3)	0.3 (0.0–1.6)	0.4 (0.0–2.1)	0.6 (0.1–2.0)	0.7 (0.1–1.7)	<0.001
Total energy intake (kcal/day), mean (SD)	2070.8 (715.0)	2099.7 (680.1)	2092.5 (664.9)	2118.6 (636.8)	2187.2 (670.8)	<0.001	2004.2 (798.2)	2110.7 (686.9)	2129.4 (658.7)	2152.0 (621.0)	2172.4 (577.8)	<0.001
Use of supplements with vitamin C (%)	11.5	15.0	18.6	20.7	23.4	<0.001	22.7	21.6	24.6	24.3	21.9	<0.05
Hypertension (%)	64.3	63.1	59.2	58.0	58.9	<0.001	55.8	58.0	60.7	62.4	66.5	<0.001
Total cholesterol, (mmol/l), mean (SD)	6.4 (1.3)	6.2 (1.2)	6.1 (1.2)	6.0 (1.2)	6.0 (1.1)	<0.001	6.0 (1.1)	6.1 (1.2)	6.1 (1.2)	6.2 (1.3)	6.3 (1.3)	<0.001
Diabetes (%)	7.1	8.9	9.4	8.9	9.3	<0.05	8.7	8.4	8.9	8.2	9.5	ns
History of CVD (%)	8.6	6.6	6.5	5.4	5.5	<0.001	6.0	6.2	6.4	6.3	7.6	ns
History of cancer (%)	4.6	5.8	5.7	6.5	7.5	<0.001	6.5	5.3	6.8	5.8	6.0	ns

Characteristics	Quintiles of beta-carotene intake (ug/day)					
	1 (low)	2	3	4	5 (high)	*p*
*Men*						
Median of vitamin intake	2897.2	4559.7	6495.0	8879.4	12,730.7	<0.001
No. of subjects	2528	2529	2529	2528	2528	–
No. of all-cause deaths	296	266	315	322	391	<0.001
No. of CVD deaths	115	100	138	143	188	<0.001
No. of cancer deaths	120	93	109	97	108	ns
Age, mean (SD)	57.4 (6.9)	57.7 (7.1)	57.9 (7.1)	58.7 (7.0)	59.5 (7.0)	<0.001
Education (%)						
Lower	43.3	39.4	37.5	38.5	38.6	<0.001
Secondary	31.4	33.9	35.7	34.2	31.4	
University	25.3	26.7	26.8	27.3	30.0	
Current smokers (%)	37.9	33.9	36.4	37.7	36.8	<0.05
BMI (kg/m^2^), mean (SD)	27.7 (4.1)	27.8 (4.2)	27.5 (4.2)	27.5 (4.1)	27.4 (4.5)	<0.05
Alcohol (g/day), median (q1–q3)	7.0 (1.0–23.5)	5.8 (0.8–20.3)	4.9 (0.7–15.4)	4.3 (0.7–15.2)	3.1 (0.4–12.0)	<0.001
Total energy intake (kcal/day), mean (SD)	2288.0 (838.1)	2321.9 (826.9)	2524.7 (799.4)	2444.7 (710.5)	2230.4 (544.9)	<0.001
Use of supplements with vitamin C (%)	13.9	14.1	13.5	11.1	11.3	<0.01
Hypertension (%)	66.6	68.9	65.0	66.6	68.0	ns
Total cholesterol (mmol/l), mean (SD)	5.8 (1.1)	5.8 (1.1)	5.8 (1.2)	5.8 (1.1)	5.9 (1.1)	<0.05
Diabetes (%)	8.9	10.1	11.1	11.1	11.1	<0.05
History of CVD (%)	10.7	11.1	11.7	13.1	17.6	<0.001
History of cancer (%)	3.1	3.5	3.1	2.5	2.4	ns
*Women*						
Median of vitamin intake	3446.9	5718.7	8206.0	10,978.2	15,033.9	<0.001
No. of subjects	2871	2869	2870	2871	2870	–
No. of all-cause deaths	160	159	154	146	162	ns
No. of CVD deaths	59	60	63	52	79	ns
No. of cancer deaths	71	59	57	55	61	ns
Age, mean (SD)	56.8 (7.0)	57.2 (7.0)	58.0 (7.0)	57.8 (7.1)	59.1 (7.0)	<0.001
Education (%)						
Lower	39.7	38.5	37.8	37.3	40.4	<0.001
Secondary	40.9	40.0	40.8	39.8	36.2	
University	19.4	21.5	21.4	22.9	23.4	
Current smokers (%)	24.4	20.8	19.9	14.8	13.0	<0.001
BMI (kg/m^2^), mean (SD)	28.2 (5.2)	28.6 (5.2)	28.9 (5.3)	29.4 (5.6)	29.8 (5.6)	<0.001
Alcohol (g/day), median (q1–q3)	0.6 (0.0–2.5)	0.5 (0.0–2.5)	0.3 (0.0–1.7)	0.4 (0.1–1.4)	0.3 (0.0–1.0)	<0.001
Total energy intake (kcal/day), mean (SD)	1940.4 (745.2)	2185.4 (726.7)	2194.8 (706.6)	2287.4 (588.1)	1960.9 (505.3)	<0.001
Use of supplements with vitamin C (%)	24.6	24.8	23.5	20.9	21.2	ns
Hypertension (%)	55.1	57.7	59.8	62.3	68.4	<0.001
Total cholesterol, (mmol/l), mean (SD)	6.0 (1.1)	6.1 (1.2)	6.1 (1.2)	6.2 (1.2)	6.3 (1.3)	<0.001
Diabetes (%)	8.2	8.5	8.5	7.9	10.6	<0.01
History of CVD (%)	5.2	5.4	6.3	7.1	8.6	<0.001
History of cancer (%)	6.3	6.5	6.2	5.0	6.2	ns

*ns* not significant, *p* > 0.05

### All-cause mortality

Gender-specific HRs and 95 % CI for all-cause mortality by quintiles of antioxidant vitamin intakes in the pooled sample are presented in Table 3. In men, inverse association between intake of vitamin C and all-cause mortality was observed but in a nonlinear manner. In the country-pooled analysis, risk of death was lower by 28 % in men in third quintile of vitamin C intake and by 20 % in fourth quintile compared with the bottom quintile. The strongest protective effect of vitamin C was seen for men in Novosibirsk (Supplementary Table I). Vitamin E intake was not associated with all-cause mortality in men, while beta-carotene consumption was associated with better survival only in men in Krakow.

**Table 3:** Age and multivariable-adjusted HR (95 % CI) of all-cause mortality in men and women according to quintiles of vitamin intakes

	Quintiles of vitamin intake				
	1 (low)	2	3	4	5 (high)
*Men*					
Vitamin C					
Model 1^a^	1	0.85 (0.73–0.98)	0.62 (0.53–0.74)	0.68 (0.58–0.80)	0.78 (0.67–0.92)
Model 2^b^	1	0.93 (0.80–1.08)	0.72 (0.61–0.85)	0.80 (0.68–0.95)	0.92 (0.78–1.09)
Vitamin E					
Model 1^a^	1	0.97 (0.83–1.15)	0.97 (0.83–1.15)	1.02 (0.87–1.20)	1.14 (0.97–1.34)
Model 2^b^	1	0.98 (0.83–1.15)	0.98 (0.83–1.15)	1.02 (0.87–1.20)	1.08 (0.92–1.27)
Beta-carotene^c^					
Model 1^a^	1	0.88 (0.70–1.11)	0.94 (0.69–1.26)	0.89 (0.70–1.14)	1.02 (0.84–1.23)
Model 2^b^	1	0.91 (0.75–1.10)	0.96 (0.69–1.33)	0.87 (0.68–1.12)	0.99 (0.83–1.17)
*Women*					
Vitamin C					
Model 1^a^	1	0.81 (0.65–1.01)	0.90 (0.72–1.12)	0.78 (0.62–0.99)	0.76 (0.60–0.97)
Model 2^b^	1	0.85 (0.68–1.06)	1.00 (0.80–1.26)	0.91 (0.72–1.15)	0.91 (0.71–1.16)
Vitamin E					
Model 1^a^	1	0.80 (0.65–0.99)	0.66 (0.52–0.83)	0.74 (0.59–0.93)	0.84 (0.66–1.06)
Model 2^b^	1	0.85 (0.69–1.06)	0.67 (0.53–0.84)	0.77 (0.61–0.97)	0.85 (0.67–1.08)
Beta-carotene					
Model 1^a^	1	0.97 (0.77–1.20)	0.87 (0.69–1.09)	0.86 (0.68–1.08)	0.88 (0.70–1.10)
Model 2^b^	1	1.03 (0.83–1.29)	0.93 (0.74–1.17)	0.93 (0.74–1.18)	0.86 (0.68–1.08)

^a^Adjusted to: age, country

^b^Adjusted to: age, country, education, smoking status, alcohol intake, BMI, hypertension, diabetes, hypercholesterolemia, history of CVD or cancer, total energy intake

^c^Significant heterogeneity between cohorts was found, see also Supplementary Table I for country-specific results

In women, in the fully adjusted model, inverse U-shaped association between intake of vitamin E and all-cause mortality was observed. Women in third quintile had 33 % less risk of mortality, and those in fourth quintile had 23 % less risk than the lowest intake group. In pooled data, vitamin C and beta-carotene intake was not associated with all-cause mortality in women.

### CVD mortality

Gender-specific HRs and 95 % CI for CVD mortality by quintiles of antioxidant vitamin are presented in Table 4. In men, in the fully adjusted model of pooled data, higher intake of vitamin C was related to a modest decrease in CVD mortality risk; the risk was lower by 35 % in third quintile and by 23 % in fourth quintile compared with the bottom group. A beneficial effect of vitamin C was particularly observed in men in Novosibirsk (Supplementary Table II). Vitamin E was not related to CVD mortality in men in pooled data. Beta-carotene intake (in second quintile) was related to the decrease in CVD mortality in men in Czech towns only (Supplementary Table II). In women, no significant associations were observed between vitamin intakes and CVD mortality.

**Table 4:** Age and multivariable-adjusted HR (95 % CI) of CVD mortality in men and women according to quintiles of vitamin intakes

	Quintiles of vitamin intake				
	1 (low)	2	3	4	5 (high)
*Men*					
Vitamin C					
Model 1^a^	1	0.90 (0.73–1.11)	0.58 (0.45–0.75)	0.66 (0.51–0.85)	0.83 (0.65–1.06)
Model 2^b^	1	0.98 (0.79–1.22)	0.65 (0.50–0.84)	0.77 (0.59–0.99)	0.94 (0.74–1.21)
Vitamin E					
Model 1^a^	1	0.95 (0.72–1.23)	0.96 (0.74–1.25)	1.09 (0.85–1.41)	1.33 (1.04–1.41)
Model 2^b^	1	0.95 (0.72–1.23)	0.93 (0.72–1.21)	1.07 (0.83–1.38)	1.20 (0.93–1.54)
Beta-carotene^c^					
Model 1^a^	1	0.74 (0.58–0.96)	0.94 (0.66–1.35)	0.93 (0.74–1.18)	1.00 (0.79–1.26)
Model 2^b^	1	0.76 (0.58–0.98)	0.95 (0.65–1.39)	0.90 (0.71–1.14)	0.92 (0.73–1.16)
*Women*					
Vitamin C					
Model 1^a^	1	0.72 (0.51–1.00)	0.84 (0.60–1.19)	0.73 (0.51–1.06)	0.72 (0.49–1.05)
Model 2^b^	1	0.75 (0.53–1.05)	0.93 (0.66–1.32)	0.86 (0.60–1.25)	0.87 (0.59–1.28)
Vitamin E					
Model 1^a^	1	0.82 (0.58–1.17)	0.82 (0.57–1.17)	0.73 (0.50–1.07)	0.81 (0.56–1.18)
Model 2^b^	1	0.86 (0.60–1.23)	0.81 (0.56–1.16)	0.76 (0.52–1.11)	0.81 (0.55–1.18)
Beta-carotene					
Model 1^a^	1	0.95 (0.66–1.36)	0.87 (0.61–1.25)	0.68 (0.46–1.00)	0.89 (0.62–1.27)
Model 2^b^	1	1.01 (0.70–1.46)	0.95 (0.66–1.37)	0.73 (0.49–1.08)	0.85 (0.59–1.23)

^a^Adjusted to: age, country

^b^Adjusted to: age, country, education, smoking status, alcohol intake, BMI, hypertension, diabetes, hypercholesterolemia, history of CVD or cancer, total energy intake

^c^Significant heterogeneity between cohorts was found, see also Supplementary Table II for country-specific results

### Cancer mortality

Cancer mortality by quintiles of antioxidant vitamins is presented in Table 5. In the fully adjusted model, there were no significant associations, neither in men, nor in women. In country-specific models in men (Supplementary Table III), vitamin C was significantly related to lower risk of cancer death by 46 and by 38 % in third quintile in Novosibirsk and in Krakow, respectively.

**Table 5:** Age and multivariable-adjusted HR (95 % CI) of cancer mortality in men and women according to quintiles of vitamin intakes

	Quintiles of vitamin intake				
	1 (low)	2	3	4	5 (high)
*Men*					
Vitamin C					
Model 1^a^	1	0.85 (0.65–1.13)	0.68 (0.51–0.92)	0.76 (0.57–1.01)	0.88 (0.66–1.16)
Model 2^b^	1	0.96 (0.73–1.26)	0.82 (0.61–1.11)	0.91 (0.68–1.23)	1.10 (0.82–1.46)
Vitamin E					
Model 1^a^	1	0.96 (0.74–1.25)	0.92 (0.71–1.21)	0.86 (0.65–1.14)	0.89 (0.67–1.18)
Model 2^b^	1	0.97 (0.75–1.26)	0.98 (0.75–1.28)	0.88 (0.67–1.17)	0.91 (0.69–1.22)
Beta-carotene					
Model 1^a^	1	0.74 (0.56–0.97)	0.90 (0.69–1.18)	0.77 (0.58–1.01)	0.84 (0.63–1.11)
Model 2^b^	1	0.78 (0.60–1.03)	0.94 (0.72–1.23)	0.80 (0.60–1.06)	0.88 (0.66–1.16)
*Women*					
Vitamin C^c^					
Model 1^a^	1	0.88 (0.62–1.25)	0.94 (0.52–1.72)	0.93 (0.51–1.71)	1.03 (0.58–1.84)
Model 2^b^	1	0.93 (0.65–1.32)	0.98 (0.59–1.65)	1.03 (0.55–1.94)	1.07 (0.59–1.93)
Vitamin E					
Model 1^a^	1	0.85 (0.60–1.19)	0.73 (0.51–1.06)	0.87 (0.60–1.26)	1.01 (0.69–1.47)
Model 2^b^	1	0.91 (0.64–1.28)	0.73 (0.51–1.06)	0.89 (0.61–1.28)	1.00 (0.69–1.47)
Beta-carotene					
Model 1^a^	1	0.84 (0.59–1.19)	0.81 (0.57–1.16)	0.88 (0.61–1.26)	0.93 (0.65–1.34)
Model 2^b^	1	0.85 (0.60–1.21)	0.83 (0.58–1.18)	0.92 (0.63–1.33)	0.93 (0.65–1.34)

^a^Adjusted to: age, country

^b^Adjusted to: age, country, education, smoking status, alcohol intake, BMI, hypertension, diabetes, hypercholesterolemia, history of CVD or cancer, total energy intake

^c^Significant heterogeneity between cohorts was found, see also Supplementary Table III for country-specific results

In our study, only 14 % of participants reported using any form of vitamin C supplementation. Exclusion of users of vitamin C supplements had no influence on the associations between antioxidant vitamins and mortality (Supplementary tables IV, V and VI). Exclusion of early deaths or those with history of CVD or cancer at baseline did not affect the results (data not shown).

## Discussion

This study, in three large urban population-based cohorts in CEE, did not find a strong evidence for a protective effect of antioxidant vitamin intake. There was some suggestion of protective effects in several subgroups (e.g., in men with moderate vitamin C intake and in women with moderate dietary intake of vitamin E), but the associations did not follow a dose–response pattern.

### Limitations and strengths of this study

There are some limitations that should be taken into account for the interpretation of our findings. First, selected samples were representative only for urban populations of Czech Republic, Russia and Poland. The overall response rate in the HAPIEE study was modest but similar to those in other contemporary cohort studies [1]. Second, we were not able to include a quantitative assessment of vitamin supplements, and the lack of such information may bias our findings. However, we controlled for vitamin C supplements use in the multivariable analyses and further stratification of the analysis according to this variable revealed in no substantial change in our results.

Third, although we controlled for the most important covariates, additional confounding factors may exist. Vitamin intake may be correlated with overall diet quality, physical activity and socioeconomical status (other than education), and we cannot exclude the possibility that the beneficial effects of vitamins may be mediated by more complex and multifactorial mechanisms. A further weakness of the study is that we did not adjust the results for the intake of saturated fatty acids; however, this effect was partially controlled by adjustment for total energy intake, which is strongly correlated with saturated fat intake. Finally, the use of FFQ may lead to an overestimation of some healthy foods, such as fruits and vegetables, and an underreporting of fats and oil intake, which in turn may result in overestimating vitamin C and beta-carotene consumption and underestimating vitamin E intake.

On the other hand, the major strength of the present study is its large size and prospective design. To the best of our knowledge, this is the first prospective study in CEE countries which has explored the relation between dietary antioxidant vitamin intakes and mortality in large cohorts. Detailed and reliable assessment of individual diet was made; moreover, FFQ data were validated against plasma biomarkers in a substudy [34]. Analyses were conducted separately for men and women. Associations stayed unchanged after exclusion of people with history of CVD or cancer at baseline, or early death (first 2 years of follow-up) or users of vitamin C supplements. Furthermore, the study was done in samples with different exposure to other cardiovascular risk factors compared to Western Europe. For example, we found high prevalence of smoking, binge drinking and unhealthy diet, and weak association between education and economic position in our cohorts [1, 2, 28, 29, 31, 33].

### Interpretation of the results

Our results are consistent to some extent with previously published observational studies. In some cohorts, dietary intake of vitamin C was inversely associated with total and CVD mortality [7, 15–17, 38–40], but other studies did not observe such association [19, 20]. Excess vitamin C consumed is excreted from the body [21, 41]. Experimental studies reported that very high concentration of vitamin C does not necessarily exert health benefits, instead may have potential harmful effects due to its pro-oxidant side effect [42]. Indeed, under certain conditions, such as the presence of free transition metals, vitamin C may function as a pro-oxidant [43]. This may explain partially that in our analysis, we did not find a dose–response effect of vitamin C intake and mortality.

Potential mechanisms that may explain the beneficial effect of vitamin C intake on mortality were postulated. Vitamin C was found to protect vascular endothelium, preventing uncontrolled vascular smooth muscle cells proliferation, and inhibit the effects of pro-inflammatory cytokines and adhesion molecules which are important in atherogenesis [4]. However, in the present study, we did not observe clear inverse associations between dietary vitamin C intake and all-cause or CVD mortality. There was an indication of protective effect of moderate intake in men, but not in women; this could be partly explained by the small number of female deaths. Further, women in our cohort had higher mean intake of vitamin C than men, and consequently, the threshold for the lowest quintile (the highest risk) was also higher. Indeed, if the same cutoff points were adopted for both sexes, the results in women were closer to results in men. However, there were differences in the number of deaths in some groups, which affected the statistical power of these analyses. In contrast to our results, in a Japanese cohort study, dietary vitamin C was related to lower risk of CVD death in women only [17]. Results from other studies on dietary vitamin C and CVD mortality are inconsistent. With a few exceptions [7, 18], cohort studies in men and women combined failed to find an association for dietary vitamin C [19, 44, 45].

In women, we observed lower risk of all-cause mortality in the groups with moderate vitamin E consumption. While in most individual cohort studies, dietary consumption of vitamin E was not related to all-cause and CVD mortality [7, 17, 19–21, 38, 44], a meta-analysis of nine prospective studies indicated that vitamin E intake may be inversely related to CHD risk in women [45]. In another systematic review, high intake of tocopherol was associated with reduction in fatal and non-fatal cardiovascular events [18]. The evidence was supported by the postulated mechanisms. Vitamin E is a group of eight structurally related isomers of which alpha-tocopherol is the most biologically active. This fat-soluble antioxidant protects lipids from peroxidation and is able to scavenge mutagenic-free radicals and inhibit the oxidation of LDL cholesterol [3, 6]. Besides its antioxidant features, tocopherols are also inhibitors of platelet aggregation and thrombus formation [46]. Observed associations between vitamin E intake and mortality in women were in line with findings of higher lipid peroxidation in women and the possibility that females may have less competing risk factors than men [45].

In the 15-year study of Dutch elderly men, higher carotenes intake was related to about 20 % lower risk of CVD death [44]. In our study, dietary beta-carotene was not associated with mortality. Similar results were found in a Danish study [20], in USA cohorts [7, 19] and in a meta-analysis of nine prospective studies [45].

Despite antioxidant vitamins being postulated to play a role in the prevention of DNA damage induced by oxidation neutralizing potentially mutagenic ROS [6], we found no associations between dietary antioxidant vitamins and cancer mortality, which is consistent with other studies [7, 19].

In the interpretation of our results, it is important to take into account of the fact that mortality rates declined, and consumption of fruits, vegetables and vegetable oils increased in CEE countries over the last two decades. In consequence, nowadays, it may be more difficult to detect any relationship. However, according to the EFSA recommendation [47], in our sample, the proportion of participants with inadequate vitamin C intake was 37 % in men and 24 % in women. Regarding vitamin E, locally recommended intake (10 mg/day for men and 8 mg/day for women [9], was not reached by about 60 % of men and 40 % of women.

Experimental studies provide convincing evidence that supplementation by antioxidant vitamins does not influence the risk of CVD and all-cause mortality. In this light, it could be argued that findings from ours and other cohort studies seem to reflect not an effect of vitamin consumption but rather confounding by unknown factors. However, it is also possible that dietary intake of vitamins may be a marker of the other important dietary ingredients, and the U-shape relationship can be explained by the fact that participants in the top quintiles had consumption of antioxidant vitamins over the recommended amounts which was not related to additional benefits.

## Conclusion

Results of the present study in CEE urban populations suggest there is no strong, dose-dependent relationship between dietary intake of vitamin C, vitamin E and beta-carotene and all-cause, CVD or cancer mortality. These findings are consistent with negative results of experimental studies on vitamin supplementation. The apparent beneficial health effects of antioxidant rich foods, which were observed in other studies, may have explanations other than antioxidant vitamin intake.

## Electronic supplementary material

Below is the link to the electronic supplementary material.

Supplementary material 1 (DOCX 26 kb)

Supplementary material 2 (DOCX 26 kb)

Supplementary material 3 (DOCX 26 kb)

Supplementary material 4 (DOCX 27 kb)

Supplementary material 5 (DOCX 28 kb)

Supplementary material 6 (DOCX 27 kb)
