# Supplementary material for: Antioxidant vitamin intake and mortality in three Central and Eastern European urban populations: the HAPIEE study
Source: Eur J Nutr. 2015 Mar 12;55(2):547–60. doi: 10.1007/s00394-015-0871-8 (PMC4767874; doi:10.1007/s00394-015-0871-8)
Supplement: Supplementary file 1 — Supplementary material 1 (DOCX 26 kb) [file 394_2015_871_MOESM1_ESM.docx]

Supplementary Table I. Age and multivariable adjusted, country-specific HR (95% CI) of all-cause mortality in men and women according to quintiles of vitamin intakes

| Vitamin | Quintiles | Czech Towns  model 1^a^ | Novosibirsk  model 1^a^ | Krakow  model 1 ^a^ | Czech Towns  model 2^b^ | Novosibirsk  model 2 ^b^ | Krakow  model 2 ^b^ |
| --- | --- | --- | --- | --- | --- | --- | --- |
| *Men* | | | | |  |  |  |
| Vitamin C | 1 | 1.00 | 1.00 | 1.00 | 1.00 | 1.00 | 1.00 |
|  | 2 | 0.97 (0.72-1.30) | 0.70 (0.55-0.88) | 0.72 (0.56-0.93) | 1.03 (0.77-1.39) | 0.77 (0.61-0.97) | 0.84 (0.65-1.09) |
|  | 3 | 0.69 (0.50-0.95) | 0.57 (0.44-0.72) | 0.63 (0.49-0.83) | 0.69 (0.50-0.95) | 0.64 (0.50-0.82) | 0.75 (0.57-0.98) |
|  | 4 | 0.96 (0.72-1.29) | 0.61 (0.48-0.78) | 0.69 (0.53-0.90) | 1.01 (0.75-1.35) | 0.71 (0.56-0.91) | 0.87 (0.67-1.13) |
|  | 5 | 1.15 (0.87-1.52) | 0.61 (0.48-0.77) | 0.73 (0.56-0.94) | 1.17 (0.88-1.56) | 0.73 (0.57-0.94) | 0.86 (0.67-1.12) |
| Vitamin E | 1 | 1.00 | 1.00 | 1.00 | 1.00 | 1.00 | 1.00 |
|  | 2 | 1.07 (0.79-1.45) | 1.05 (0.82-1.34) | 0.91 (0.69-1.20) | 1.05 (0.78-1.43) | 1.05 (0.82-1.35) | 0.94 (0.71-1.23) |
|  | 3 | 0.95 (0.70-1.30) | 0.93 (0.72-1.21) | 0.99 (0.76-1.29) | 1.00 (0.73-1.37) | 0.97 (0.75-1.25) | 1.00 (0.76-1.31) |
|  | 4 | 1.03 (0.76-1.38) | 0.97 (0.75-1.25) | 0.99 (0.75-1.30) | 0.99 (0.73-1.34) | 0.98 (0.76-1.26) | 1.01 (0.77-1.32) |
|  | 5 | 1.07 (0.80-1.43) | 1.25 (0.98-1.58) | 1.03 (0.79-1.34) | 1.00 (0.74-1.34) | 1.21 (0.95-1.54) | 1.03 (0.79-1.35) |
| Beta-carotene | 1 | 1.00 | 1.00 | 1.00 | 1.00 | 1.00 | 1.00 |
|  | 2 | 0.81 (0.59-1.11) | 1.09 (0.84-1.41) | 0.77 (0.59-1.00) | 0.83 (0.61-1.15) | 1.08 (0.84-1.40) | 0.80 (0.61-1.05) |
|  | 3 | 0.94 (0.70-1.28) | 1.20 (0.93-1.54) | 0.72 (0.55-0.94) | 0.92 (0.67-1.24) | 1.28 (0.99-1.67) | 0.75 (0.57-0.98) |
|  | 4 | 1.07 (0.80-1.43) | 0.95 (0.73-1.24) | 0.71 (0.54-0.92) | 1.04 (0.77-1.39) | 0.95 (0.73-1.24) | 0.69 (0.53-0.90) |
|  | 5 | 1.08 (0.81-1.44) | 1.16 (0.90-1.49) | 0.85 (0.66-1.10) | 1.05 (0.79-1.40) | 1.11 (0.86-1.43) | 0.83 (0.65-1.08) |
| *Women* | | | | |  |  |  |
| Vitamin C | 1 | 1.00 | 1.00 | 1.00 | 1.00 | 1.00 | 1.00 |
|  | 2 | 0.89 (0.61-1.29) | 0.66 (0.45-0.97) | 0.96 (0.67-1.36) | 0.92 (0.63-1.34) | 0.68 (0.47-1.00) | 1.06 (0.74-1.51) |
|  | 3 | 0.64 (0.43-0.97) | 0.76 (0.52-1.12) | 0.97 (0.68-1.38) | 0.72 (0.48-1.10) | 0.81 (0.55-1.18) | 1.04 (0.73-1.49) |
|  | 4 | 0.90 (0.62-1.31) | 1.00 (0.70-1.44) | 0.81 (0.56-1.17) | 1.02 (0.70-1.48) | 1.11 (0.77-1.60) | 0.94 (0.65-1.37) |
|  | 5 | 0.70 (0.47-1.05) | 0.86 (0.59-1.25) | 0.85 (0.59-1.23) | 0.69 (0.46-1.03) | 0.95 (0.65-1.40) | 1.02 (0.70-1.49) |
| Vitamin E | 1 | 1.00 | 1.00 | 1.00 | 1.00 | 1.00 | 1.00 |
|  | 2 | 1.11 (0.76-1.63) | 0.83 (0.56-1.23) | 0.79 (0.57-1.10) | 1.16 (0.79-1.70) | 0.87 (0.59-1.29) | 0.83 (0.59-1.15) |
|  | 3 | 0.78 (0.52-1.18) | 0.85 (0.58-1.25) | 0.65 (0.45-0.92) | 0.78 (0.52-1.19) | 0.93 (0.63-1.38) | 0.73 (0.51-1.04) |
|  | 4 | 0.75 (0.49-1.13) | 1.08 (0.75-1.57) | 0.53 (0.36-0.77) | 0.82 (0.54-1.24) | 1.11 (0.76-1.61) | 0.59 (0.41-0.87) |
|  | 5 | 0.86 (0.58-1.27) | 1.00 (0.69-1.45) | 0.62 (0.43-0.89) | 0.87 (0.59-1.29) | 1.04 (0.71-1.51) | 0.67 (0.47-0.96) |
| Beta-carotene | 1 | 1.00 | 1.00 | 1.00 | 1.00 | 1.00 | 1.00 |
|  | 2 | 0.86 (0.57-1.29) | 1.09 (0.75-1.58) | 0.87 (0.60-1.25) | 0.86 (0.58-1.29) | 1.14 (0.78-1.66) | 1.00 (0.69-1.45) |
|  | 3 | 0.97 (0.66-1.42) | 0.57 (0.36-0.89) | 0.98 (0.69-1.39) | 0.98 (0.67-1.44) | 0.61 (0.38-0.97) | 1.03 (0.72-1.47) |
|  | 4 | 0.79 (0.53-1.18) | 0.93 (0.63-1.36) | 0.89 (0.62-1.28) | 0.82 (0.55-1.23) | 0.91 (0.62-1.34) | 0.93 (0.65-1.34) |
|  | 5 | 0.76 (0.51-1.13) | 1.02 (0.70-1.49) | 0.71 (0.48-1.03) | 0.84 (0.56-1.26) | 0.92 (0.63-1.34) | 0.76 (0.52-1.11) |

^a^ adjusted to: age

^b^ adjusted to: age, education, smoking status, alcohol intake, BMI, hypertension, diabetes, hypercholesterolemia, history of CVD or cancer, total energy intake
