# Supplementary material for: Antioxidant vitamin intake and mortality in three Central and Eastern European urban populations: the HAPIEE study
Source: Eur J Nutr. 2015 Mar 12;55(2):547–60. doi: 10.1007/s00394-015-0871-8 (PMC4767874; doi:10.1007/s00394-015-0871-8)
Supplement: Supplementary file 2 — Supplementary material 2 (DOCX 26 kb) [file 394_2015_871_MOESM2_ESM.docx]

Supplementary Table II. Age and multivariable adjusted, country-specific HR (95% CI) of CVD mortality in men and women according to quintile of vitamin intakes

| Vitamin | Quintiles | Czech Towns  model 1^a^ | Novosibirsk  model 1 ^a^ | Krakow  model 1 ^a^ | Czech Towns  model 2^b^ | Novosibirsk  model 2 ^b^ | Krakow  model 2 ^b^ |
| --- | --- | --- | --- | --- | --- | --- | --- |
| *Men* |  |  |  |  |  |  |  |
| Vitamin C | 1 | 1.00 | 1.00 | 1.00 | 1.00 | 1.00 | 1.00 |
|  | 2 | 0.83 (0.53-1.32) | 0.73 (0.53-1.00) | 0.89 (0.56-1.40) | 0.85 (0.53-1.35) | 0.78 (0.57-1.06) | 1.05 (0.67-1.66) |
|  | 3 | 0.44 (0.25-0.75) | 0.65 (0.47-0.90) | 0.72 (0.45-1.16) | 0.40 (0.23-0.70) | 0.71 (0.51-0.99) | 0.86 (0.53-1.40) |
|  | 4 | 0.73 (0.46-1.16) | 0.65 (0.47-0.90) | 0.78 (0.49-1.25) | 0.77 (0.48-1.23) | 0.71 (0.51-0.99) | 1.01 (0.63-1.62) |
|  | 5 | 0.94 (0.60-1.45) | 0.61 (0.44-0.85) | 1.18 (0.77-1.80) | 0.88 (0.56-1.37) | 0.70 (0.50-0.98) | 1.32 (0.86-2.04) |
| Vitamin E | 1 | 1.00 | 1.00 | 1.00 | 1.00 | 1.00 | 1.00 |
|  | 2 | 0.46 (0.27-0.79) | 0.88 (0.62-1.24) | 0.94 (0.56-1.56) | 0.43 (0.25-0.74) | 0.85 (0.60-1.21) | 1.01 (0.60-1.68) |
|  | 3 | 0.65 (0.41-1.05) | 0.98 (0.70-1.37) | 1.22 (0.76-1.96) | 0.68 (0.42-1.10) | 1.00 (0.71-1.41) | 1.23 (0.76-1.98) |
|  | 4 | 0.65 (0.41-1.04) | 0.98 (0.70-1.38) | 1.17 (0.72-1.91) | 0.61 (0.38-0.97) | 0.97 (0.69-1.36) | 1.17 (0.72-1.91) |
|  | 5 | 0.82 (0.53-1.26) | 1.27 (0.92-1.75) | 1.64 (1.04-2.57) | 0.70 (0.45-1.08) | 1.16 (0.84-1.60) | 1.63 (1.03-2.56) |
| Beta-carotene | 1 | 1.00 | 1.00 | 1.00 | 1.00 | 1.00 | 1.00 |
|  | 2 | 0.54 (0.31-0.95) | 0.86 (0.60-1.24) | 0.72 (0.45-1.15) | 0.56 (0.32-0.98) | 0.86 (0.60-1.23) | 0.75 (0.47-1.21) |
|  | 3 | 0.94 (0.58-1.52) | 1.22 (0.87-1.72) | 0.67 (0.42-1.08) | 0.87 (0.54-1.42) | 1.29 (0.92-1.83) | 0.69 (0.43-1.12) |
|  | 4 | 1.08 (0.68-1.71) | 0.88 (0.62-1.27) | 0.89 (0.58-1.37) | 1.01 (0.63-1.60) | 0.87 (0.60-1.24) | 0.85 (0.55-1.32) |
|  | 5 | 0.96 (0.60-1.53) | 1.07 (0.76-1.50) | 0.93 (0.60-1.42) | 0.85 (0.53-1.37) | 1.00 (0.71-1.41) | 0.87 (0.56-1.34) |
| *Women* |  |  |  |  |  |  |  |
| Vitamin C | 1 | 1.00 | 1.00 | 1.00 | 1.00 | 1.00 | 1.00 |
|  | 2 | 1.00 (0.53-1.90) | 0.67 (0.41-1.08) | 0.90 (0.48-1.70) | 0.95 (0.50-1.81) | 0.67 (0.41-1.10) | 1.01 (0.54-1.91) |
|  | 3 | 0.78 (0.39-1.55) | 0.58 (0.34-0.99) | 1.05 (0.57-1.92) | 0.86 (0.43-1.71) | 0.64 (0.37-1.09) | 1.11 (0.60-2.04) |
|  | 4 | 0.79 (0.40-1.57) | 0.79 (0.48-1.30) | 0.95 (0.51-1.76) | 0.87 (0.44-1.73) | 0.89 (0.54-1.48) | 1.13 (0.60-2.11) |
|  | 5 | 0.77 (0.39-1.53) | 0.66 (0.39-1.11) | 0.86 (0.45-1.65) | 0.71 (0.36-1.43) | 0.76 (0.44-1.29) | 1.03 (0.53-2.00) |
| Vitamin E | 1 | 1.00 | 1.00 | 1.00 | 1.00 | 1.00 | 1.00 |
|  | 2 | 1.03 (0.54-1.98) | 0.93 (0.57-1.52) | 0.72 (0.40-1.30) | 1.06 (0.55-2.04) | 1.01 (0.62-1.65) | 0.70 (0.38-1.27) |
|  | 3 | 0.73 (0.36-1.47) | 0.61 (0.35-1.06) | 0.68 (0.37-1.27) | 0.71 (0.35-1.45) | 0.71 (0.40-1.24) | 0.79 (0.42-1.49) |
|  | 4 | 0.66 (0.32-1.36) | 0.87 (0.52-1.45) | 0.70 (0.38-1.29) | 0.74 (0.36-1.53) | 0.91 (0.54-1.54) | 0.80 (0.43-1.48) |
|  | 5 | 0.86 (0.45-1.65) | 0.84 (0.51-1.39) | 0.73 (0.40-1.34) | 0.87 (0.45-1.67) | 0.92 (0.55-1.54) | 0.77 (0.42-1.44) |
| Beta-carotene | 1 | 1.00 | 1.00 | 1.00 | 1.00 | 1.00 | 1.00 |
|  | 2 | 0.65 (0.30-1.39) | 1.03 (0.62-1.72) | 0.82 (0.45-1.52) | 0.63 (0.30-1.35) | 1.13 (0.68-1.88) | 0.96 (0.51-1.79) |
|  | 3 | 1.11 (0.59-2.10) | 0.54 (0.29-1.01) | 0.74 (0.40-1.38) | 1.15 (0.61-2.16) | 0.62 (0.32-1.17) | 0.80 (0.43-1.50) |
|  | 4 | 1.07 (0.57-2.01) | 0.86 (0.51-1.46) | 0.66 (0.35-1.26) | 1.18 (0.63-2.23) | 0.89 (0.53-1.51) | 0.70 (0.37-1.34) |
|  | 5 | 0.48 (0.22-1.04) | 0.91 (0.54-1.52) | 0.74 (0.41-1.36) | 0.53 (0.24-1.16) | 0.83 (0.49-1.39) | 0.81 (0.44-1.50) |

^a^ adjusted to: age

^b^ adjusted to: age, education, smoking status, alcohol intake, BMI, hypertension, diabetes, hypercholesterolemia, history of CVD or cancer, total energy intake
