# Supplementary material for: Antioxidant vitamin intake and mortality in three Central and Eastern European urban populations: the HAPIEE study
Source: Eur J Nutr. 2015 Mar 12;55(2):547–60. doi: 10.1007/s00394-015-0871-8 (PMC4767874; doi:10.1007/s00394-015-0871-8)
Supplement: Supplementary file 3 — Supplementary material 3 (DOCX 26 kb) [file 394_2015_871_MOESM3_ESM.docx]

Supplementary Table III. Age and multivariable adjusted, country-specific HR (95% CI) of cancer mortality in men and women according to quintiles of vitamin intakes

| Vitamin | Quintiles | Czech Towns  model 1^a^ | Novosibirsk  model 1 ^a^ | Krakow  model 1 ^a^ | Czech Towns  model 2^b^ | Novosibirsk  model 2 ^b^ | Krakow  model 2 ^b^ |
| --- | --- | --- | --- | --- | --- | --- | --- |
| *Men* |  |  |  |  |  |  |  |
| Vitamin C | 1 | 1.00 | 1.00 | 1.00 | 1.00 | 1.00 | 1.00 |
|  | 2 | 0.77 (0.48-1.24) | 0.68 (0.41-1.13) | 0.62 (0.41-0.94) | 0.86 (0.53-1.40) | 0.75 (0.46-1.25) | 0.75 (0.49-1.14) |
|  | 3 | 0.73 (0.45-1.18) | 0.48 (0.27-0.84) | 0.50 (0.32-0.78) | 0.77 (0.48-1.25) | 0.54 (0.31-0.95) | 0.62 (0.40-0.97) |
|  | 4 | 0.93 (0.60-1.46) | 0.71 (0.43-1.17) | 0.66 (0.44-0.99) | 0.98 (0.62-1.53) | 0.84 (0.51-1.40) | 0.85 (0.56-1.29) |
|  | 5 | 1.17 (0.76-1.78) | 1.00 (0.63-1.57) | 0.60 (0.39-0.91) | 1.30 (0.84-2.00) | 1.25 (0.79-1.99) | 0.77 (0.50-1.18) |
| Vitamin E | 1 | 1.00 | 1.00 | 1.00 | 1.00 | 1.00 | 1.00 |
|  | 2 | 1.46 (0.92-2.31) | 1.16 (0.70-1.93) | 0.81 (0.52-1.25) | 1.47 (0.93-2.34) | 1.19 (0.72-1.98) | 0.82 (0.53-1.28) |
|  | 3 | 1.02 (0.62-1.67) | 0.96 (0.57-1.64) | 1.00 (0.66-1.50) | 1.08 (0.66-1.78) | 1.01 (0.59-1.72) | 1.03 (0.68-1.55) |
|  | 4 | 1.06 (0.66-1.72) | 0.93 (0.55-1.59) | 0.84 (0.54-1.30) | 1.03 (0.63-1.67) | 0.97 (0.56-1.66) | 0.92 (0.59-1.42) |
|  | 5 | 0.99 (0.61-1.60) | 1.30 (0.79-2.13) | 0.63 (0.39-1.01) | 0.97 (0.60-1.59) | 1.32 (0.80-2.18) | 0.67 (0.42-1.08) |
| Beta-carotene | 1 | 1.00 | 1.00 | 1.00 | 1.00 | 1.00 | 1.00 |
|  | 2 | 0.93 (0.59-1.47) | 1.17 (0.71-1.96) | 1.00 (0.65-1.54) | 0.97 (0.61-1.53) | 1.14 (0.68-1.90) | 1.07 (0.70-1.66) |
|  | 3 | 0.76 (0.47-1.22) | 1.00 (0.59-1.71) | 0.91 (0.59-1.40) | 0.75 (0.46-1.23) | 1.04 (0.61-1.80) | 0.95 (0.61-1.47) |
|  | 4 | 0.93 (0.60-1.46) | 1.05 (0.62-1.77) | 0.65 (0.41-1.03) | 0.94 (0.60-1.47) | 1.02 (0.60-1.72) | 0.65 (0.41-1.04) |
|  | 5 | 0.89 (0.57-1.38) | 0.92 (0.54-1.57) | 0.84 (0.55-1.30) | 0.92 (0.59-1.45) | 0.87 (0.50-1.50) | 0.90 (0.58-1.39) |
| *Women* |  |  |  |  |  |  |  |
| Vitamin C | 1 | 1.00 | 1.00 | 1.00 | 1.00 | 1.00 | 1.00 |
|  | 2 | 0.86 (0.50-1.48) | 1.14 (0.50-2.57) | 0.80 (0.46-1.41) | 0.89 (0.51-1.53) | 1.21 (0.53-2.76) | 0.85 (0.49-1.50) |
|  | 3 | 0.53 (0.28-1.00) | 1.68 (0.78-3.62) | 1.02 (0.60-1.72) | 0.61 (0.32-1.15) | 1.65 (0.76-3.58) | 1.04 (0.62-1.77) |
|  | 4 | 0.92 (0.54-1.57) | 1.79 (0.83-3.86) | 0.55 (0.29-1.03) | 1.05 (0.61-1.81) | 1.96 (0.90-4.29) | 0.58 (0.31-1.09) |
|  | 5 | 0.77 (0.44-1.35) | 2.09 (0.99-4.42) | 0.79 (0.45-1.40) | 0.75 (0.43-1.33) | 2.18 (1.02-4.65) | 0.87 (0.49-1.55) |
| Vitamin E | 1 | 1.00 | 1.00 | 1.00 | 1.00 | 1.00 | 1.00 |
|  | 2 | 1.12 (0.63-1.98) | 0.73 (0.34-1.56) | 0.92 (0.55-1.55) | 1.22 (0.69-2.16) | 0.72 (0.34-1.55) | 1.00 (0.59-1.70) |
|  | 3 | 0.96 (0.53-1.72) | 1.07 (0.54-2.12) | 0.63 (0.35-1.14) | 0.96 (0.53-1.72) | 1.08 (0.54-2.17) | 0.69 (0.38-1.24) |
|  | 4 | 0.74 (0.40-1.37) | 0.91 (0.44-1.88) | 0.55 (0.30-1.02) | 0.80 (0.43-1.50) | 0.90 (0.43-1.86) | 0.60 (0.32-1.11) |
|  | 5 | 0.96 (0.54-1.70) | 1.16 (0.59-2.28) | 0.81 (0.47-1.40) | 0.97 (0.55-1.72) | 1.12 (0.56-2.21) | 0.83 (0.48-1.44) |
| Beta-carotene | 1 | 1.00 | 1.00 | 1.00 | 1.00 | 1.00 | 1.00 |
|  | 2 | 1.00 (0.56-1.78) | 1.26 (0.57-2.77) | 0.71 (0.39-1.29) | 1.00 (0.56-1.78) | 1.26 (0.57-2.81) | 0.75 (0.41-1.37) |
|  | 3 | 0.91 (0.51-1.62) | 0.95 (0.40-2.24) | 0.95 (0.55-1.64) | 0.90 (0.50-1.61) | 0.93 (0.39-2.22) | 0.95 (0.55-1.64) |
|  | 4 | 0.90 (0.50-1.59) | 1.59 (0.75-3.38) | 0.85 (0.48-1.48) | 0.86 (0.48-1.53) | 1.51 (0.71-3.24) | 0.85 (0.48-1.51) |
|  | 5 | 0.84 (0.47-1.50) | 1.85 (0.88-3.86) | 0.69 (0.39-1.24) | 0.92 (0.51-1.66) | 1.66 (0.79-3.49) | 0.68 (0.38-1.23) |

^a^ adjusted to: age

^b^ adjusted to: age, education, smoking status, alcohol intake, BMI, hypertension, diabetes, hypercholesterolemia, history of CVD or cancer, total energy intake
