# Supplementary material for: Antioxidant vitamin intake and mortality in three Central and Eastern European urban populations: the HAPIEE study
Source: Eur J Nutr. 2015 Mar 12;55(2):547–60. doi: 10.1007/s00394-015-0871-8 (PMC4767874; doi:10.1007/s00394-015-0871-8)
Supplement: Supplementary file 4 — Supplementary material 4 (DOCX 27 kb) [file 394_2015_871_MOESM4_ESM.docx]

Supplementary Table IV. Age and multivariable adjusted, country-specific and total HR (95% CI) of all-cause mortality in men and women according to quintiles of vitamin intakes. Results for participants who take no vitamin supplements regularly (n = 23022).

| Vitamin | Quintiles | Czech Towns  model 1^a^ | Novosibirsk  model 1 ^a^ | Krakow  model 1 ^a^ | Total  model 1^a,b^ | Czech Towns  model 2^c^ | Novosibirsk  model 2 ^c^ | Krakow  model 2 ^c^ | Total  model 2^b,c^ |
| --- | --- | --- | --- | --- | --- | --- | --- | --- | --- |
| *Men* | | | | | | | | | |
| Vitamin C | 1 | 1.00 | 1.00 | 1.00 | 1.00 | 1.00 | 1.00 | 1.00 | 1.00 |
|  | 2 | 0.86 (0.63-1.18) | 0.72 (0.57-0.91) | 0.72 (0.54-0.94) | 0.80 (0.69-0.93) | 0.92 (0.67-1.26) | 0.79 (0.62-1.00) | 0.85 (0.65-1.13) | 0.89 (0.76-1.04) |
|  | 3 | 0.56 (0.40-0.80) | 0.57 (0.45-0.74) | 0.68 (0.52-0.90) | 0.60 (0.50-0.71) | 0.56 (0.40-0.80) | 0.65 (0.50-0.84) | 0.81 (0.61-1.07) | 0.69 (0.58-0.82) |
|  | 4 | 0.81 (0.59-1.11) | 0.63 (0.49-0.81) | 0.70 (0.53-0.92) | 0.69 (0.58-0.82) | 0.84 (0.61-1.16) | 0.75 (0.58-0.96) | 0.91 (0.69-1.21) | 0.82 (0.69-0.98) |
|  | 5 | 1.08 (0.81-1.45) | 0.64 (0.50-0.82) | 0.77 (0.58-1.00) | 0.75 (0.63-0.89) | 1.13 (0.84-1.52) | 0.76 (0.59-0.97) | 0.92 (0.70-1.21) | 0.89 (0.74-1.05) |
| Vitamin E | 1 | 1.00 | 1.00 | 1.00 | 1.00 | 1.00 | 1.00 | 1.00 | 1.00 |
|  | 2 | 1.03 (0.74-1.43) | 1.04 (0.81-1.35) | 0.85 (0.64-1.14) | 0.95 (0.80-1.13) | 1.02 (0.73-1.42) | 1.05 (0.81-1.36) | 0.86 (0.65-1.15) | 0.95 (0.80-1.13) |
|  | 3 | 0.89 (0.64-1.25) | 0.92 (0.71-1.20) | 0.89 (0.67-1.18) | 0.91 (0.77-1.08) | 0.96 (0.68-1.35) | 0.96 (0.74-1.25) | 0.90 (0.68-1.19) | 0.92 (0.77-1.09) |
|  | 4 | 0.97 (0.70-1.34) | 0.99 (0.76-1.28) | 0.89 (0.67-1.18) | 1.02 (0.86-1.20) | 0.94 (0.68-1.30) | 1.01 (0.78-1.31) | 0.91 (0.68-1.21) | 1.02 (0.86-1.21) |
|  | 5 | 1.06 (0.77-1.45) | 1.27 (0.99-1.62) | 0.99 (0.75-1.31) | 1.14 (0.94-1.32) | 1.02 (0.74-1.40) | 1.23 (0.96-1.57) | 1.02 (0.77-1.35) | 1.06 (0.89-1.26) |
| Beta-carotene^d^ | 1 | 1.00 | 1.00 | 1.00 | 1.00 | 1.00 | 1.00 | 1.00 | 1.00 |
|  | 2 | 0.78 (0.55-1.10) | 1.09 (0.84-1.43) | 0.64 (0.48-0.86) | 0.85 (0.63-1.14) | 0.82 (0.58-1.16) | 1.10 (0.84-1.43) | 0.68 (0.51-0.91) | 0.85 (0.64-1.14) |
|  | 3 | 0.99 (0.71-1.38) | 1.24 (0.96-1.61) | 0.68 (0.51-0.89) | 0.95 (0.67-1.35) | 0.97 (0.70-1.35) | 1.33 (1.02-1.74) | 0.70 (0.53-0.93) | 0.97 (0.66-1.43) |
|  | 4 | 1.14 (0.83-1.56) | 0.94 (0.71-1.24) | 0.62 (0.47-0.81) | 0.87 (0.60-1.27) | 1.11 (0.81-1.52) | 0.95 (0.72-1.25) | 0.61 (0.46-0.81) | 0.86 (0.61-1.21) |
|  | 5 | 1.02 (0.75-1.40) | 1.19 (0.92-1.54) | 0.75 (0.57-0.97) | 0.97 (0.75-1.26) | 1.00 (0.73-1.37) | 1.15 (0.88-1.50) | 0.75 (0.58-0.98) | 0.95 (0.73-1.23) |
| *Women* | | | | | | | | | |
| Vitamin C | 1 | 1.00 | 1.00 | 1.00 | 1.00 | 1.00 | 1.00 | 1.00 | 1.00 |
|  | 2 | 0.79 (0.51-1.21) | 0.73 (0.49-1.08) | 0.93 (0.64-1.37) | 0.76 (0.60-0.97) | 0.80 (0.52-1.23) | 0.76 (0.51-1.13) | 1.06 (0.72-1.56) | 0.81 (0.64-1.03) |
|  | 3 | 0.60 (0.38-0.96) | 0.66 (0.43-1.00) | 0.84 (0.56-1.25) | 0.81 (0.64-1.04) | 0.64 (0.40-1.02) | 0.70 (0.46-1.06) | 0.92 (0.62-1.37) | 0.91 (0.71-1.17) |
|  | 4 | 0.84 (0.55-1.28) | 0.94 (0.64-1.37) | 0.75 (0.50-1.13) | 0.72 (0.56-0.94) | 0.98 (0.64-1.51) | 1.03 (0.70-1.52) | 0.86 (0.57-1.30) | 0.84 (0.65-1.09) |
|  | 5 | 0.65 (0.41-1.02) | 0.85 (0.58-1.27) | 0.76 (0.50-1.15) | 0.70 (0.54-0.92) | 0.66 (0.42-1.04) | 0.95 (0.64-1.42) | 0.92 (0.60-1.40) | 0.86 (0.66-1.13) |
| Vitamin E | 1 | 1.00 | 1.00 | 1.00 | 1.00 | 1.00 | 1.00 | 1.00 | 1.00 |
|  | 2 | 1.17 (0.74-1.84) | 0.77 (0.51-1.15) | 0.72 (0.50-1.04) | 0.76 (0.60-0.96) | 1.22 (0.77-1.93) | 0.80 (0.53-1.20) | 0.73 (0.51-1.05) | 0.80 (0.63-1.02) |
|  | 3 | 0.90 (0.56-1.45) | 0.77 (0.51-1.15) | 0.52 (0.34-0.78) | 0.67 (0.52-0.86) | 0.90 (0.56-1.45) | 0.85 (0.56-1.29) | 0.59 (0.39-0.89) | 0.68 (0.52-0.87) |
|  | 4 | 0.89 (0.55-1.43) | 0.97 (0.66-1.42) | 0.58 (0.39-0.86) | 0.68 (0.53-0.89) | 0.95 (0.59-1.53) | 1.01 (0.68-1.49) | 0.65 (0.43-0.96) | 0.71 (0.54-0.92) |
|  | 5 | 1.01 (0.64-1.59) | 0.90 (0.61-1.34) | 0.52 (0.34-0.78) | 0.81 (0.63-1.05) | 1.02 (0.64-1.61) | 0.93 (0.62-1.38) | 0.55 (0.36-0.84) | 0.82 (0.63-1.06) |
| Beta-carotene | 1 | 1.00 | 1.00 | 1.00 | 1.00 | 1.00 | 1.00 | 1.00 | 1.00 |
|  | 2 | 0.85 (0.53-1.37) | 1.09 (0.73-1.60) | 0.80 (0.53-1.21) | 0.91 (0.71-1.16) | 0.85 (0.53-1.37) | 1.14 (0.77-1.69) | 0.90 (0.60-1.38) | 0.96 (0.75-1.23) |
|  | 3 | 0.93 (0.59-1.46) | 0.60 (0.37-0.95) | 1.05 (0.71-1.53) | 0.92 (0.72-1.18) | 1.01 (0.64-1.58) | 0.64 (0.40-1.03) | 1.10 (0.75-1.62) | 0.99 (0.77-1.27) |
|  | 4 | 0.89 (0.57-1.40) | 0.95 (0.64-1.42) | 0.81 (0.54-1.21) | 0.82 (0.63-1.06) | 0.89 (0.56-1.40) | 0.95 (0.63-1.41) | 0.83 (0.55-1.25) | 0.88 (0.68-1.14) |
|  | 5 | 0.80 (0.51-1.27) | 0.96 (0.64-1.43) | 0.60 (0.39-0.93) | 0.81 (0.63-1.05) | 0.91 (0.57-1.45) | 0.87 (0.58-1.30) | 0.65 (0.42-1.00) | 0.79 (0.61-1.02) |

^a^ adjusted to: age

^b^ pooled sample adjusted for country

^c^ adjusted to: age, education, smoking status, alcohol intake, BMI, hypertension, diabetes, hypercholesterolemia, history of CVD or cancer, total energy intake

^d^  significant heterogeneity between cohorts
