# Supplementary material for: Antioxidant vitamin intake and mortality in three Central and Eastern European urban populations: the HAPIEE study
Source: Eur J Nutr. 2015 Mar 12;55(2):547–60. doi: 10.1007/s00394-015-0871-8 (PMC4767874; doi:10.1007/s00394-015-0871-8)
Supplement: Supplementary file 5 — Supplementary material 5 (DOCX 28 kb) [file 394_2015_871_MOESM5_ESM.docx]

Supplementary Table V. Age and multivariable adjusted, country-specific and total HR (95% CI) of CVD mortality in men and women according to quintiles of vitamin intakes. Results for participants who take no vitamin supplements regularly (n = 23022).

| Vitamin | Quintiles | Czech Towns  model 1^a^ | Novosibirsk  model 1 ^a^ | Krakow  model 1 ^a^ | Total  model 1^a,b^ | Czech Towns  model 2^c^ | Novosibirsk  model 2 ^c^ | Krakow  model 2 ^c^ | Total  model 2^b,c^ |
| --- | --- | --- | --- | --- | --- | --- | --- | --- | --- |
| *Men* | | | | | | | | | |
| Vitamin C | 1 | 1.00 | 1.00 | 1.00 | 1.00 | 1.00 | 1.00 | 1.00 | 1.00 |
|  | 2 | 0.79 (0.49-1.27) | 0.74 (0.54-1.02) | 1.05 (0.64-1.72) | 0.86 (0.69-1.08) | 0.78 (0.48-1.27) | 0.78 (0.56-1.07) | 1.30 (0.79-2.15) | 0.95 (0.76-1.19) |
|  | 3 | 0.34 (0.18-0.63) | 0.66 (0.47-0.93) | 0.87 (0.52-1.45) | 0.56 (0.43-0.74) | 0.31 (0.17-0.58) | 0.72 (0.51-1.01) | 1.05 (0.63-1.77) | 0.63 (0.48-0.83) |
|  | 4 | 0.62 (0.37-1.02) | 0.66 (0.47-0.92) | 0.83 (0.49-1.40) | 0.69 (0.53-0.90) | 0.64 (0.38-1.08) | 0.72 (0.51-1.01) | 1.13 (0.66-1.92) | 0.80 (0.61-1.05) |
|  | 5 | 0.82 (0.51-1.31) | 0.67 (0.48-0.94) | 1.55 (0.98-2.45) | 0.80 (0.62-1.04) | 0.79 (0.49-1.28) | 0.74 (0.52-1.03) | 1.84 (1.16-2.93) | 0.91 (0.70-1.18) |
| Vitamin E | 1 | 1.00 | 1.00 | 1.00 | 1.00 | 1.00 | 1.00 | 1.00 | 1.00 |
|  | 2 | 0.45 (0.25-0.78) | 0.91 (0.64-1.30) | 0.92 (0.53-1.60) | 0.90 (0.68-1.20) | 0.42 (0.24-0.75) | 0.89 (0.62-1.27) | 0.98 (0.56-1.70) | 0.90 (0.68-1.20) |
|  | 3 | 0.61 (0.36-1.01) | 1.01 (0.71-1.43) | 1.19 (0.71-1.99) | 0.97 (0.73-1.27) | 0.65 (0.39-1.09) | 1.05 (0.74-1.49) | 1.19 (0.71-2.00) | 0.93 (0.71-1.23) |
|  | 4 | 0.52 (0.31-0.87) | 1.01 (0.71-1.42) | 1.24 (0.74-2.07) | 1.09 (0.84-1.43) | 0.48 (0.29-0.81) | 1.00 (0.71-1.42) | 1.23 (0.73-2.06) | 1.08 (0.83-1.41) |
|  | 5 | 0.72 (0.46-1.15) | 1.31 (0.94-1.81) | 1.73 (1.07-2.79) | 1.28 (0.99-1.66) | 0.65 (0.40-1.04) | 1.19 (0.86-1.66) | 1.76 (1.08-2.85) | 1.14 (0.88-1.49) |
| Beta-carotene^d^ | 1 | 1.00 | 1.00 | 1.00 | 1.00 | 1.00 | 1.00 | 1.00 | 1.00 |
|  | 2 | 0.56 (0.31-1.00) | 0.83 (0.58-1.21) | 0.61 (0.37-1.01) | 0.70 (0.54-0.92) | 0.60 (0.33-1.07) | 0.84 (0.58-1.21) | 0.65 (0.39-1.08) | 0.73 (0.56-0.95) |
|  | 3 | 0.85 (0.50-1.42) | 1.22 (0.87-1.72) | 0.63 (0.38-1.04) | 0.92 (0.63-1.37) | 0.80 (0.48-1.35) | 1.30 (0.91-1.84) | 0.66 (0.40-1.09) | 0.91 (0.59-1.40) |
|  | 4 | 1.01 (0.62-1.64) | 0.88 (0.61-1.26) | 0.86 (0.55-1.34) | 0.90 (0.71-1.15) | 0.95 (0.58-1.56) | 0.86 (0.60-1.25) | 0.84 (0.54-1.31) | 0.88 (0.68-1.12) |
|  | 5 | 0.78 (0.47-1.29) | 1.09 (0.78-1.53) | 0.81 (0.51-1.27) | 0.93 (0.73-1.18) | 0.72 (0.43-1.20) | 1.03 (0.72-1.45) | 0.78 (0.49-1.24) | 0.88 (0.69-1.12) |
| *Women* | | | | | | | | | |
| Vitamin C | 1 | 1.00 | 1.00 | 1.00 | 1.00 | 1.00 | 1.00 | 1.00 | 1.00 |
|  | 2 | 0.73 (0.34-1.56) | 0.79 (0.48-1.30) | 0.89 (0.45-1.77) | 0.69 (0.48-0.99) | 0.67 (0.31-1.43) | 0.82 (0.50-1.36) | 1.05 (0.53-2.10) | 0.73 (0.51-1.05) |
|  | 3 | 0.73 (0.34-1.56) | 0.46 (0.25-0.85) | 0.89 (0.45-1.77) | 0.74 (0.50-1.08) | 0.75 (0.35-1.62) | 0.51 (0.27-0.95) | 0.93 (0.47-1.86) | 0.82 (0.56-1.21) |
|  | 4 | 0.81 (0.39-1.70) | 0.79 (0.47-1.33) | 1.01 (0.52-1.97) | 0.75 (0.51-1.11) | 0.92 (0.44-1.96) | 0.91 (0.54-1.55) | 1.19 (0.61-2.32) | 0.88 (0.60-1.31) |
|  | 5 | 0.65 (0.30-1.42) | 0.68 (0.39-1.18) | 0.63 (0.29-1.38) | 0.62 (0.40-0.95) | 0.67 (0.30-1.46) | 0.79 (0.45-1.40) | 0.77 (0.35-1.70) | 0.77 (0.50-1.19) |
| Vitamin E | 1 | 1.00 | 1.00 | 1.00 | 1.00 | 1.00 | 1.00 | 1.00 | 1.00 |
|  | 2 | 0.88 (0.40-1.92) | 0.98 (0.59-1.60) | 0.69 (0.35-1.35) | 0.82 (0.55-1.21) | 0.93 (0.42-2.03) | 1.05 (0.63-1.73) | 0.61 (0.31-1.21) | 0.84 (0.56-1.24) |
|  | 3 | 0.85 (0.40-1.84) | 0.45 (0.24-0.85) | 0.58 (0.28-1.20) | 0.84 (0.57-1.24) | 0.87 (0.40-1.88) | 0.53 (0.28-1.01) | 0.64 (0.31-1.35) | 0.82 (0.55-1.22) |
|  | 4 | 0.75 (0.33-1.67) | 0.81 (0.47-1.39) | 0.90 (0.47-1.70) | 0.69 (0.45-1.06) | 0.81 (0.36-1.81) | 0.88 (0.51-1.51) | 0.97 (0.51-1.86) | 0.70 (0.46-1.07) |
|  | 5 | 0.81 (0.38-1.72) | 0.77 (0.45-1.32) | 0.66 (0.32-1.34) | 0.78 (0.52-1.18) | 0.85 (0.40-1.83) | 0.84 (0.49-1.46) | 0.65 (0.32-1.34) | 0.76 (0.50-1.15) |
| Beta-carotene | 1 | 1.00 | 1.00 | 1.00 | 1.00 | 1.00 | 1.00 | 1.00 | 1.00 |
|  | 2 | 0.66 (0.28-1.54) | 0.96 (0.57-1.63) | 0.68 (0.34-1.34) | 0.89 (0.61-1.32) | 0.61 (0.26-1.45) | 1.07 (0.63-1.82) | 0.73 (0.37-1.47) | 0.93 (0.63-1.38) |
|  | 3 | 0.84 (0.39-1.81) | 0.57 (0.30-1.06) | 0.73 (0.38-1.41) | 0.80 (0.54-1.19) | 0.94 (0.44-2.04) | 0.65 (0.34-1.24) | 0.77 (0.39-1.49) | 0.88 (0.59-1.30) |
|  | 4 | 1.16 (0.57-2.35) | 0.80 (0.46-1.39) | 0.46 (0.21-0.98) | 0.58 (0.38-0.89) | 1.28 (0.62-2.60) | 0.84 (0.49-1.46) | 0.46 (0.21-0.99) | 0.60 (0.39-0.93) |
|  | 5 | 0.53 (0.23-1.24) | 0.79 (0.46-1.37) | 0.61 (0.31-1.20) | 0.87 (0.53-1.16) | 0.58 (0.25-1.38) | 0.72 (0.41-1.25) | 0.62 (0.31-1.23) | 0.74 (0.50-1.09) |

^a^ adjusted to age

^b^ pooled sample adjusted for country

^c^ adjusted to: age, education, smoking status, alcohol intake, BMI, hypertension, diabetes, hypercholesterolemia, history of CVD or cancer, total energy intake

^d^  significant heterogeneity between cohorts
