# Supplementary material for: Antioxidant vitamin intake and mortality in three Central and Eastern European urban populations: the HAPIEE study
Source: Eur J Nutr. 2015 Mar 12;55(2):547–60. doi: 10.1007/s00394-015-0871-8 (PMC4767874; doi:10.1007/s00394-015-0871-8)
Supplement: Supplementary file 6 — Supplementary material 6 (DOCX 27 kb) [file 394_2015_871_MOESM6_ESM.docx]

Supplementary Table VI. Age and multivariable adjusted, country-specific and total HR (95% CI) of cancer mortality in men and women according to quintiles of vitamin intakes. Results for participants who take no vitamin supplements regularly (n = 23022).

| Vitamin | Quintiles | Czech Towns  model 1^a^ | Novosibirsk  model 1 ^a^ | Krakow  model 1 ^a^ | Total  model 1^a,b^ | Czech Towns  model 2^c^ | Novosibirsk  model 2 ^c^ | Krakow  model 2 ^c^ | Total  model 2^b,c^ |
| --- | --- | --- | --- | --- | --- | --- | --- | --- | --- |
| *Men* | | | | | | | | | |
| Vitamin C | 1 | 1.00 | 1.00 | 1.00 | 1.00 | 1.00 | 1.00 | 1.00 | 1.00 |
|  | 2 | 0.62 (0.37-1.04) | 0.72 (0.44-1.20) | 0.59 (0.38-0.92) | 0.78 (0.59-1.05) | 0.69 (0.41-1.17) | 0.81 (0.49-1.35) | 0.71 (0.46-1.12) | 0.89 (0.66-1.19) |
|  | 3 | 0.63 (0.38-1.06) | 0.48 (0.27-0.86) | 0.51 (0.33-0.81) | 0.62 (0.46-0.85) | 0.68 (0.41-1.14) | 0.55 (0.31-0.99) | 0.63 (0.40-1.00) | 0.76 (0.55-1.04) |
|  | 4 | 0.82 (0.51-1.31) | 0.74 (0.45-1.23) | 0.70 (0.46-1.06) | 0.79 (0.59-1.07) | 0.85 (0.52-1.37) | 0.92 (0.55-1.54) | 0.93 (0.61-1.42) | 0.98 (0.73-1.33) |
|  | 5 | 1.18 (0.77-1.82) | 0.97 (0.61-1.56) | 0.52 (0.33-0.83) | 0.84 (0.63-1.13) | 1.37 (0.88-2.13) | 1.23 (0.76-1.99) | 0.67 (0.42-1.06) | 1.07 (0.79-1.44) |
| Vitamin E | 1 | 1.00 | 1.00 | 1.00 | 1.00 | 1.00 | 1.00 | 1.00 | 1.00 |
|  | 2 | 1.45 (0.88-2.37) | 1.12 (0.67-1.89) | 0.73 (0.46-1.15) | 0.92 (0.70-1.22) | 1.45 (0.89-2.38) | 1.14 (0.67-1.91) | 0.73 (0.46-1.15) | 0.93 (0.70-1.22) |
|  | 3 | 0.94 (0.55-1.61) | 0.96 (0.56-1.65) | 0.85 (0.55-1.31) | 0.86 (0.65-1.14) | 1.01 (0.59-1.74) | 0.98 (0.57-1.69) | 0.88 (0.57-1.36) | 0.91 (0.69-1.21) |
|  | 4 | 1.14 (0.69-1.89) | 0.97 (0.56-1.66) | 0.79 (0.51-1.23) | 0.86 (0.64-1.14) | 1.12 (0.68-1.87) | 1.01 (0.59-1.74) | 0.87 (0.55-1.36) | 0.89 (0.67-1.18) |
|  | 5 | 0.95 (0.56-1.59) | 1.26 (0.76-2.09) | 0.59 (0.37-0.96) | 0.83 (0.62-1.12) | 0.94 (0.56-1.60) | 1.27 (0.76-2.12) | 0.65 (0.40-1.05) | 0.85 (0.63-1.14) |
| Beta-carotene | 1 | 1.00 | 1.00 | 1.00 | 1.00 | 1.00 | 1.00 | 1.00 | 1.00 |
|  | 2 | 0.90 (0.54-1.48) | 1.24 (0.73-2.09) | 0.83 (0.53-1.29) | 0.76 (0.57-1.01) | 0.93 (0.56-1.54) | 1.21 (0.72-2.05) | 0.90 (0.57-1.42) | 0.81 (0.61-1.07) |
|  | 3 | 0.85 (0.51-1.41) | 1.08 (0.63-1.86) | 0.82 (0.53-1.27) | 0.99 (0.75-1.30) | 0.85 (0.51-1.42) | 1.13 (0.65-1.96) | 0.87 (0.56-1.36) | 1.02 (0.77-1.34) |
|  | 4 | 1.05 (0.65-1.68) | 1.06 (0.61-1.82) | 0.53 (0.32-0.85) | 0.73 (0.55-0.99) | 1.04 (0.65-1.69) | 1.05 (0.61-1.81) | 0.54 (0.33-0.87) | 0.77 (0.57-1.03) |
|  | 5 | 0.92 (0.57-1.48) | 0.96 (0.55-1.66) | 0.74 (0.47-1.15) | 0.83 (0.62-1.11) | 0.94 (0.58-1.52) | 0.92 (0.53-1.61) | 0.81 (0.52-1.27) | 0.86 (0.64-1.16) |
| *Women* | | | | | | | | | |
| Vitamin C^d^ | 1 | 1.00 | 1.00 | 1.00 | 1.00 | 1.00 | 1.00 | 1.00 | 1.00 |
|  | 2 | 0.77 (0.41-1.43) | 1.13 (0.48-2.66) | 0.66 (0.35-1.24) | 0.85 (0.58-1.24) | 0.76 (0.40-1.42) | 1.17 (0.49-2.76) | 0.71 (0.38-1.33) | 0.81 (0.54-1.20) |
|  | 3 | 0.51 (0.25-1.03) | 1.46 (0.64-3.33) | 0.87 (0.49-1.55) | 0.87 (0.41-1.83) | 0.55 (0.27-1.12) | 1.39 (0.60-3.20) | 0.90 (0.50-1.62) | 0.86 (0.53-1.39) |
|  | 4 | 0.82 (0.44-1.51) | 1.74 (0.78-3.88) | 0.46 (0.22-0.93) | 0.85 (0.47-1.56) | 0.98 (0.53-1.83) | 1.78 (0.79-4.01) | 0.48 (0.24-0.99) | 0.93 (0.46-1.86) |
|  | 5 | 0.75 (0.40-1.40) | 1.88 (0.85-4.17) | 0.73 (0.39-1.35) | 0.99 (0.53-1.85) | 0.73 (0.39-1.37) | 1.88 (0.84-4.22) | 0.79 (0.42-1.48) | 0.98 (0.57-1.67) |
| Vitamin E | 1 | 1.00 | 1.00 | 1.00 | 1.00 | 1.00 | 1.00 | 1.00 | 1.00 |
|  | 2 | 1.03 (0.53-2.01) | 0.56 (0.24-1.30) | 0.82 (0.47-1.44) | 0.72 (0.49-1.06) | 1.10 (0.56-2.15) | 0.54 (0.23-1.27) | 0.88 (0.50-1.55) | 0.78 (0.53-1.14) |
|  | 3 | 0.96 (0.50-1.87) | 1.07 (0.53-2.16) | 0.43 (0.21-0.86) | 0.67 (0.45-1.00) | 0.92 (0.47-1.80) | 1.05 (0.51-2.15) | 0.48 (0.24-0.97) | 0.67 (0.45-1.01) |
|  | 4 | 0.76 (0.37-1.54) | 0.81 (0.37-1.75) | 0.58 (0.31-1.09) | 0.73 (0.48-1.10) | 0.80 (0.39-1.63) | 0.80 (0.37-1.73) | 0.64 (0.34-1.22) | 0.74 (0.49-1.12) |
|  | 5 | 1.08 (0.57-2.04) | 1.09 (0.54-2.21) | 0.59 (0.31-1.11) | 0.87 (0.57-1.31) | 1.05 (0.55-1.98) | 1.03 (0.50-2.10) | 0.62 (0.33-1.18) | 0.87 (0.57-1.32) |
| Beta-carotene | 1 | 1.00 | 1.00 | 1.00 | 1.00 | 1.00 | 1.00 | 1.00 | 1.00 |
|  | 2 | 1.15 (0.58-2.28) | 1.36 (0.57-3.23) | 0.60 (0.31-1.16) | 0.77 (0.52-1.15) | 1.14 (0.57-2.28) | 1.34 (0.56-3.23) | 0.64 (0.33-1.26) | 0.79 (0.53-1.18) |
|  | 3 | 1.07 (0.54-2.13) | 1.07 (0.42-2.69) | 0.91 (0.51-1.63) | 0.94 (0.64-1.38) | 1.12 (0.56-2.23) | 1.01 (0.40-2.59) | 0.92 (0.51-1.67) | 0.97 (0.66-1.42) |
|  | 4 | 1.15 (0.59-2.25) | 1.94 (0.86-4.38) | 0.70 (0.38-1.32) | 0.78 (0.51-1.18) | 1.02 (0.52-2.00) | 1.89 (0.83-4.28) | 0.71 (0.38-1.34) | 0.81 (0.53-1.24) |
|  | 5 | 1.01 (0.51-2.01) | 2.04 (0.91-4.57) | 0.54 (0.28-1.05) | 0.94 (0.63-1.40) | 1.13 (0.56-2.26) | 1.89 (0.84-4.28) | 0.56 (0.28-1.10) | 0.95 (0.63-1.42) |

^a^ adjusted to age

^b^ pooled sample adjusted for country

^c^ adjusted to: age, education, smoking status, alcohol intake, BMI, hypertension, diabetes, hypercholesterolemia, history of CVD or cancer, total energy intake

^d^  significant heterogeneity between cohorts
